# Artificial Intelligence-Enabled Prescribing in Opioid Use Disorder Care: Decision Support, Risk Scores, Large Language Model Copilots, and Digital Therapeutics (2024–2025)

**DOI:** 10.1159/ear/acrag006

**Published:** 2026-09-18

**Authors:** Mike Zack, Valentin Skryabin

**Affiliations:** a PGxAI Inc., Palo Alto, CA, USA; b Royal College of Psychiatrists, London, UK

**Keywords:** Opioid use disorder, Medications for opioid use disorder, Clinical decision support systems, Electronic health records, Prescription drug monitoring program, European AI Act, Digital therapeutics

## Abstract

**Background:**

This narrative, practice-oriented review maps the 2024–2025 landscape of artificial intelligence (AI)-enabled prescribing in opioid use disorder care, where the evidence base is most developed, while noting cautious extension to other substance use disorders.

**Summary:**

We link concrete clinical decisions to fit-for-purpose tools across four domains: electronic health record (EHR)-embedded prediction and safety nudges; prescription drug monitoring program (PDMP)-derived analytics and risk scores; clinician-facing large language model (LLM) copilots; and prescription digital therapeutics, just-in-time adaptive interventions (JITAIs), and personal sensing systems. The regulatory analysis uses US frameworks as worked examples and translates the same governance questions to European settings through the EU AI Act, MDR/IVDR medical software requirements, GDPR, the European Health Data Space, and the HMA-EMA data and AI work plan. Evidence maturity is uneven. EHR-based clinical decision support for naloxone coprescribing and protocolized buprenorphine initiation has the most established implementation base. PDMP risk scores are clinically visible but incompletely validated and should remain advisory. Digital therapeutics and JITAI approaches may support engagement and retention, but they should not automate dosing.

**Key Messages:**

LLM copilots are most defensible as audited, citation-first conformance checkers rather than autonomous prescribers; patient-facing agents should be restricted to education, skills support, navigation, and escalation. Across settings, the highest-yield systems are transparent, narrowly scoped, workflow-native, and evaluated with prescribing-centered outcomes such as dose optimization, 28/90/180-day retention, naloxone coprescribing and fills, and overdose-related safety. We propose safeguards that keep AI explainable, equitable, transferable across health systems, and subordinate to clinical judgment.

## Introduction

Medication decisions in substance use disorders (SUDs) are increasingly moving from trial-and-error toward transparent, auditable, artificial intelligence (AI)-supported workflows [1–10]. The evidence base, however, is not evenly distributed across all SUDs. In 2024–2025, the most developed prescribing evidence concerns opioid use disorder (OUD): medications for opioid use disorder (MOUD), buprenorphine initiation and titration, naloxone coprescribing, risk review, and follow-up in high-tolerance contexts. We therefore frame this review around OUD-related prescribing while using digital therapeutics, just-in-time adaptive intervention (JITAI), and conversational agent literature on other substances only where it informs engagement, monitoring, or between-visit support [1–10].

This article is a narrative and practical review, not a systematic review or meta-analysis. We used targeted, iterative searches of peer-reviewed literature and official sources, emphasizing 2024–2025 developments while retaining seminal earlier work where needed for implementation, evaluation, or governance. Sources included clinical studies, implementation reports, regulatory and standards documents, policy materials, and selected commercial or news sources when they were necessary to document product status, market transitions, or regulatory debate. Peer-reviewed and official regulatory sources were prioritized for normative claims; vendor and news materials were used descriptively and interpreted cautiously.

The review links concrete prescribing questions – who needs naloxone, how to initiate buprenorphine safely in the fentanyl era, when to intensify follow-up, how to interpret prescription drug monitoring program (PDMP) signals, and how to use large language models (LLMs) without unsafe autonomy to fit-for-purpose AI tools. US frameworks remain useful because they provide operational examples of device/nondevice clinical decision support (CDS) boundaries, certified health IT transparency, and opioid treatment program (OTP) documentation. For *European Addiction Research* readers, we treat these as comparators and add European regulatory and implementation translation through the EU AI Act, MDR/IVDR, GDPR, the European Health Data Space (EHDS), and HMA-EMA data and AI governance [11–29].

Our central claim is pragmatic: the most consistently reported gains come from interventions that are narrowly scoped, workflow-native, and legible – tools that reduce friction and standardize safe actions – paired with governance that clarifies boundaries and builds equity-minded oversight [30–33]. In practice, the highest-yield uses are less about discovering new treatment rules and more about making existing safe practices reliably executable: surfacing the right checklist at the right moment, prepopulating the right documentation, and turning guideline-aligned actions into default pathways while retaining explicit escape hatches for clinician judgment [30–33].

## Regulatory and Clinical Context: Why Software Now Mediates Prescribing

Two forces make governance and workflow design nonnegotiable [34–36]. First, clinical practice has changed: illicit fentanyl’s high potency and tissue retention have altered buprenorphine induction dynamics and shifted dose-response expectations toward higher, individualized targets in many patients [34–36]. In Europe, OUD treatment also sits within heterogeneous national systems, with opioid agonist treatment coverage and delivery models varying substantially by country and with emerging concern about synthetic opioids, including nitazenes [27, 28]. Second, regulation has changed. US OTP modernization and HTI-1 transparency rules now define where and how decision support can sit inside care delivery, while European law increasingly emphasizes risk classification, medical device software qualification, data protection, interoperability, and human oversight [11–29].

European readers should not read the US regulatory discussion as a proposed template. The relevant translation is functional. US rules ask whether software supports or directs clinical judgment; whether the basis for a recommendation is independently reviewable; and whether certified health IT exposes the data, evidence, and risk management attributes behind a decision support intervention (DSI) [14–19]. In Europe, analogous questions arise under the EU AI Act for high-risk AI systems, under MDR/IVDR and MDCG software guidance when a system qualifies as medical device software, under GDPR and EHDS for health data processing and interoperability, and under the HMA-EMA Network Data Steering Group work plan for medicines regulation data and AI governance [20–26].

In February 2024, HHS/SAMHSA finalized major updates to OTP standards under 42 CFR Part 8 (effective April 2, 2024; compliance by October 2, 2024) [11–13]. The rule broadens telehealth options for evaluation and initiation pathways, updates take-home methadone schedules, and codifies flexibilities that create new points where standardized, auditable support can reduce variation without displacing clinician judgment [11–13, 37–39]. Telehealth screening and admission workflows can be structured to recommend when audio-only assessment is adequate for buprenorphine initiation versus when video or in-person evaluation is warranted – particularly for methadone, where visual assessment helps mitigate sedation risk [11–13]. The updated take-home framework replaces rigid “stability” heuristics with individualized clinical judgment and documentation, creating a niche for tools that make criteria explicit, generate defensible documentation, and track safeguards (storage education, packaging, diversion controls) without turning risk scoring into denial [12, 13, 40, 41]. A practical corollary is that “documentation burden” becomes a safety variable: when documentation is difficult, clinicians may either avoid indicated care or under-document clinical reasoning, increasing both clinical and regulatory risk. Workflow-native tools that produce clear, reviewable notes can therefore be safety infrastructure, not administrative clutter, provided they preserve clinician authorship and do not launder opaque scores into seemingly objective determinations [12, 13, 16, 33].

The European implementation problem is different. There is no single EU analog of US PDMPs, and prescribing, dispensing, pharmacy, and national health record data are organized differently across member states. EHDS may improve cross-border data exchange and secondary use over time, but near-term deployments must assume variable data completeness, different legal bases for processing, language and workflow heterogeneity, and local standards for opioid agonist treatment delivery [23–28]. This makes external validation, local calibration, and transparent disclosure of data provenance more important than importing a US-trained model or a proprietary risk score without context.

Clinical reality in the fentanyl era further heightens the value of protocolized decision support [35, 42–44]. Observational data suggest improved retention at higher buprenorphine maintenance doses (e.g., 24 mg/day vs. 16 mg/day in some cohorts), and emergency departments have operationalized high-dose day-1 pathways (“macrodosing”) under monitoring with low precipitated withdrawal (PW) rates reported in protocolized settings [35, 42–44]. Low-dose/overlap initiation (LDI, “microdosing”) remains useful for selected patients – those unable to tolerate abstinence, with prior PW, transitioning from methadone or prescribed full agonists, or strongly preferring overlap – but real-world cohorts show it is not PW-proof and operational constraints matter [45–48]. In short, the “right” induction strategy is increasingly a branching, setting-dependent decision – exactly the kind of choice that benefits from workflow-native, transparent logic and guardrails rather than opaque prediction [36, 43, 49]. The clinical aim is not to force all patients into one induction pathway, but to ensure that each pathway – standard, high-dose with monitoring, and LDI – is triggered by visible criteria, paired with explicit safety steps, and recorded in a way that is auditable and reproducible across clinicians and sites [36, 43, 49].

## US and European Governance Spine for AI-Enabled Decision Support

US regulation draws a consequential line between CDS that aids a clinician’s reasoning and software that functions as a medical device [14, 15]. Under the 21st Century Cures Act framework and FDA guidance, a CDS function is generally outside the device definition only when it (i) does not analyze medical images/signals, (ii) displays or analyzes medical information, (iii) supports rather than directs health-care professional decisions, and (iv) enables independent review of the basis for recommendations so clinicians do not rely primarily on the software [14, 15]. Functions that provide patient-specific risk probabilities or time-critical directives often fall on the device side of this boundary, particularly when the rationale is not independently reviewable [15]. In SUD contexts, overdose risk models, PDMP-derived risk scores, or tools that effectively function as “do not prescribe” gates should therefore be assumed to trigger device-level expectations unless carefully rescoped to be transparent, reviewable, and nondirective [14, 15].

FDA’s lifecycle expectations for AI-enabled device software have sharpened, including total product lifecycle expectations and content for marketing submissions, aligned with Good Machine Learning Practice and international transparency principles [50–53]. FDA’s Predetermined Change Control Plan (PCCP) framework enables controlled post-market model updates when modifications, protocols, and impact assessment are prespecified [51]. In high-stakes prescribing, the practical point is that “model drift” is not a theoretical concern: drug supply changes, care pathways change, and the meaning of common proxies changes, so governance must treat updates as a normal part of safety operations rather than as a rare engineering event [33, 51, 52].

In parallel, ONC’s HTI-1 final rule modernizes health IT certification by replacing the legacy CDS criterion with DSIs [16–19, 54]. Starting January 1, 2025, health IT developers with health IT certified to the DSI criterion must comply with associated maintenance requirements, after updating previously certified CDS functionality to meet the DSI criterion and providing it to customers by December 31, 2024 [16–19, 54]. For predictive DSIs supplied by the health IT developer as part of certified modules, HTI-1 also requires intervention risk management practices and a public summary [16–18, 55, 56]. A key implementation implication is that transparency must be available at the moment of decision: if the information about data inputs, limitations, error tradeoffs, or subgroup performance is buried in a PDF, it cannot meaningfully counter automation bias at the bedside or in the clinic [16–19, 33, 54].

European governance points in the same practical direction but uses different legal architecture. The EU AI Act classifies AI systems that may pose serious risks to health, safety, or fundamental rights as high risk and requires risk management, data governance, technical documentation, logging, transparency to deployers, human oversight, robustness, accuracy, and cybersecurity [20, 21]. For clinical AI that is itself medical device software or a safety component of a regulated product, MDR/IVDR qualification and classification remain central, with MDCG guidance clarifying how software functions are assessed [22]. GDPR and EHDS add privacy, data minimization, lawful basis, patient control, interoperability, and secure secondary-use obligations that matter directly for EHR-derived prediction, sensing, and learning health-system pipelines [23–25].

For European addiction services, the practical design principle is to separate assistive context from predictive or directive functions in the same way the US device/nondevice boundary encourages. Guideline retrieval, checklisting, documentation support, and source-grounded conformance checking should remain visibly reviewable. Patient-specific risk probabilities, automated triage thresholds, dose suggestions, or refusal gates require a much higher governance burden: defined intended use, traceability, local validation, human oversight, version control, post-deployment monitoring, and rollback criteria [14–26, 33].

Together, FDA and ONC point to a design principle: separate “assistive context” (guideline retrieval, checklists, documentation support) from “predictive or directive” functions (risk scoring, patient-specific probabilities, dosing directives) and ensure the basis for any recommendation is visible and reviewable at the point of care [14–16, 57]. This is not merely regulatory hygiene; it reduces automation bias, preserves clinician discretion, and enables auditability in a high-stigma domain [33, 58, 59]. The practical stance is to treat prediction as a trigger for review, not a substitute for it: prediction can route attention, but protocol and clinician reasoning should determine action, with explicit documentation of the clinical rationale rather than a numerical score [14–16, 33, 58, 59] (Table 1).

**Table 1.** AI-enabled tools for OUD prescribing and adjunctive care

Tool category	Prescribing or care use case	Evidence maturity	Prescribing-related outcomes to measure	Main risks	Recommended safeguards
EHR-based CDS, order sets, and safety nudges	Naloxone co-prescribing; buprenorphine initiation pathways; dose and safety checklists; documentation support	Most implementation-ready; strongest evidence for narrowly targeted, workflow-native prompts and order sets	Naloxone orders and fills; MOUD initiation; dose optimization; retention at 28/90/180 days; alert acceptance/override; overdose-related events	Alert fatigue; poor trigger specificity; hidden rationale; reduction in indicated prescribing if risk labels are misused	High-specificity triggers; one-click actions; visible eligibility logic; override capture; subgroup monitoring; monthly safety review
Population risk panels	Team-based review of very-high-risk patients and routing to mitigation bundles	Moderate implementation evidence; effects depend on ownership and actionability	Completed risk review; naloxone access; PDMP/medication reconciliation; care coordination; mortality and serious safety events	Surveillance without intervention; stale risk labels; inequitable targeting	Bind each risk tier to a named owner and action bundle; audit completion; recalibrate and stratify outcomes
PDMP-derived analytics and risk scores	Review of dispensing history, overlap, multiple prescribers/pharmacies, and potential safety signals	High visibility but uneven validation for hard clinical endpoints	Appropriate review completion; opioid-benzodiazepine overlap; documentation quality; access to indicated analgesia/MOUD; adverse events	Automation bias; false high-risk or low-risk reassurance; stigmatizing labels; data gaps such as missing OTP methadone	Keep scores advisory; show underlying transactions; prohibit threshold-based denial; disclose limitations; monitor subgroup effects
Prescription digital therapeutics and JITAI/personal sensing	Between-visit engagement; skills practice; contingency management; early outreach; symptom or craving-triggered contact	Mixed and evolving; stronger for engagement than for direct prescribing decisions	Engagement; adherence; refill timeliness; retention; outreach completion; patient-reported symptoms; non-prescribed opioid use	Missing-not-at-random data; prompt habituation; privacy burden; overreach into dosing	Use signals to prioritize contact, not automate dosing; consent and data minimization; treat missingness as a possible safety signal
LLM clinician copilots	Chart summarization; guideline retrieval; draft documentation; protocol conformance checking; missing safety step detection	Promising but early; requires scenario testing and monitoring	Unsafe action rate; unsupported claim rate; omission rate; clinician corrections; time saved; auditability	Hallucinations; omissions; outdated sources; automation bias; unclear accountability	Retrieval-first design; mandatory citations; audit trails; model versioning; clinician review before action; refusal/escalation rules
Patient-facing conversational agents	Psychoeducation; motivational support; coping skills; adherence reminders; navigation to services	Mixed evidence; safer for support than individualized clinical decisions	Engagement; self-efficacy; service linkage; adherence reminders; escalation events; patient experience	Misdiagnosis; individualized dosing advice; crisis mismanagement; privacy and language risks	No diagnosis or dosing; crisis-language detection; warm handoff; approved content library; clear scope disclosure
Respiratory safety and overdose alert technologies	Desaturation/respiratory monitoring; alert escalation; responder routing; adjunctive harm reduction	Early and device-specific; limited clinical endpoint evidence	Alert accuracy; responder arrival; naloxone availability/use; false alarms; patient acceptability; overdose-related safety	Sensor drift; missingness; false reassurance; polysubstance confounding	Layered safety plan; human response pathway; device lifecycle monitoring; naloxone education and access

AI, artificial intelligence; CDS, clinical decision support; EHR, electronic health record; JITAI, just-in-time adaptive intervention; LLM, large language model; MOUD, medications for opioid use disorder; OTP, opioid treatment program; PDMP, prescription drug monitoring program.

### Tool Class 1: EHR-Embedded Predictors, Order Sets, and Safety Nudges

Among the tool classes reviewed here, EHR-embedded CDS has the clearest implementation evidence for changing prescribing behavior when the intervention is narrow, visible, and linked to a frictionless action [30, 31, 60–63]. EHR-embedded decision support has the strongest and most reproducible evidence base for shifting prescribing behaviors, especially when tools are narrowly targeted and paired with frictionless actions [30, 60]. A consistent pattern emerges: high-specificity triggers tied to clear guidelines or statutes, paired with one-click orders and minimal documentation burden, can substantially increase evidence-aligned “secondary prescribing,” particularly naloxone coprescribing [31, 61–63]. ED and inpatient implementations show similar directionality, with adoption dependent on trigger precision and alert fatigue management [30, 31]. Where these tools fail, the failure is often not clinical disagreement but ergonomics: too many low-value firings, unclear eligibility logic, and no straightforward path from alert to action [30, 31, 60]. Thus, optimizing signal-to-noise and lowering action friction can be more influential than adding more variables or more complex modeling [30, 31, 60].

Population-level risk panels can also concentrate attention on the “very-high-risk tail” [1, 2]. The VA’s STORM dashboard illustrates a model where risk stratification routes patients to interdisciplinary review and mitigation bundles (e.g., naloxone, PDMP review, care coordination) [1, 2]. Evaluations suggest benefits in mandated review contexts (including mortality signals in some analyses), though effects on specific serious adverse events can be variable, reinforcing the need for careful outcome specification and time horizons [64, 65]. A practical deployment principle is to bind stratification to specific actions and ownership: if a high-risk label does not map to a defined workflow (who reviews, what mitigations are available, what constitutes completion), risk panels can become surveillance without intervention [1, 2, 30].

For MOUD initiation, EHR tools are most useful when they operationalize protocols rather than produce opaque recommendations [66–68]. ED platforms such as EMBED and successors illustrate that usability, workflow fit, and “default-with-exceptions” design may matter as much as clinical content [66–68]. A pragmatic implementation approach is to present pathway “cards” for standard induction, high-dose induction under monitoring, and LDI – autopopulated from the chart (withdrawal scores, last opioid timing, naloxone administration, sedative co-use, relevant vitals) and linked to one-click order sets for dosing, adjunctive medications, discharge supplies, naloxone, and documentation [36, 43, 49, 68]. The strongest design choice is transparency: eligibility criteria, guardrails (sedation checks, observation windows, contraindication flags), and explicit fallbacks should be visible to clinicians, creating an auditable record of why a strategy was selected [14, 16, 30]. This supports safe scaling in mixed experience environments where clinicians rotate, coverage is fragmented, and the same patient may traverse ED, inpatient, and outpatient settings; in those conditions, consistent protocol execution often matters as much as the protocol itself [30, 66–68].

### Tool Class 2: PDMP-Derived Analytics-High Visibility, Uneven Validation

PDMPs are ubiquitous at the point of prescribing, and many jurisdictions encourage or require clinician review [69]. In practice, many PDMP interfaces present vendor-generated analytics, including composite risk scores and alerts derived solely from dispensing patterns (numbers of prescribers/pharmacies, MME, overlap, recency) [70–72]. These scores are highly visible and can influence clinician behavior, especially when integrated into EHR workflows via single sign-on (e.g., PMP Gateway), which increases PDMP query rates and can reduce specific risky patterns such as opioid-benzodiazepine overlaps [4, 73]. The operational appeal is obvious: a single number compresses a complex history. The danger is equally obvious: compression can erase context, convert correlation into implied causation, and shift a clinician from reviewing data to reacting to a label [58, 59, 74].

The central limitation is validation [59, 74]. Authoritative guidance cautions that PDMP-generated composite risk scores have limited independent validation for predicting hard clinical outcomes (e.g., overdose) and should not replace clinical judgment [59, 74]. Independent validation is limited and methodologically heterogeneous; one commonly cited study comparing a PDMP narcotic score to WHO ASSIST found only fair agreement and notable false-positive/false-negative rates at chosen thresholds, highlighting the risk of inappropriate restriction or missed risk [75, 76]. Data coverage gaps can further distort inferences; for example, methadone dispensed by OTPs may be absent due to confidentiality/reporting constraints, creating systematic blind spots [72, 74, 77]. These limitations matter because PDMP-derived scores can be used in contested situations – acute pain, chronic pain, transitions from incarceration, or contested histories – where a false “high risk” classification can lead to undertreatment, disengagement, or moral injury, while a false “low risk” can provide false reassurance [59, 74].

Because PDMP scores compress heterogeneous clinical and social signals into a single label, they can prime automation bias and epistemic injustice if treated as dispositive [58, 59]. A defensible posture is to treat PDMP analytics as advisory triage signals that must be corroborated by underlying dispensing details and clinical context, with documentation explicitly separating clinical rationale from the numeric score [69, 74]. Where PDMP-derived scores are embedded into certified EHR modules, HTI-1 transparency requirements provide a governance scaffold: validation setting, operating points and error rates, subgroup performance/fairness, limitations, and maintenance plans should be visible and kept current [17, 18, 54]. Clinics should avoid blanket thresholds that function as denials of indicated therapy and should implement local monitoring of firing rates, acceptance/override behavior, and subgroup differences [16, 33, 78]. This monitoring should treat “override” as a potentially informative signal rather than a compliance failure: frequent overrides may indicate miscalibrated thresholds, poorly specified clinical intent, or a mismatch between the score’s training context and the clinic’s population [16, 33, 78].

Regulatory debate reflects these tensions [79–81]. Petitions have urged FDA to classify and regulate proprietary PDMP scoring as device software; FDA responses underscore a case-by-case posture rather than blanket enforcement [79–81]. Vendor materials emphasize that scores are advisory and should not be used as the sole justification to provide or refuse medications, but ethical concerns remain when tools are operationalized as hard thresholds in practice [59, 74, 82]. Therefore, governance should explicitly prohibit translating a PDMP score into a “no care” default; instead, high scores should trigger structured review steps that focus on reconciling the PDMP record with the patient narrative and clinical findings [59, 74, 82].

For European settings, the PDMP discussion is best understood as a cautionary analogy rather than a direct policy recommendation. National prescription registers, e-prescribing systems, pharmacy claims, or shared medication records can support safer prescribing, but their use should emphasize reviewable transaction data and clinical reconciliation rather than proprietary composite labels. Where cross-border care, private dispensing, or fragmented records create blind spots, the safest design is to disclose data coverage explicitly and avoid converting incomplete data into denial thresholds [20–28, 69, 74].

### Tool Class 3: Prescription Digital Therapeutics and JITAI-Between-Visit Stabilization

Prescription digital therapeutics such as reSET and reSET-O have a defined operating model (structured CBT/CRA-based content, skills practice, contingency management) and are positioned as adjuncts to standard care [6, 83]. Following Pear Therapeutics’ bankruptcy and asset transfer, a virtual care provider reacquired and relaunched these PDTs within telehealth workflows and payer arrangements [84–87]. Trial and real-world analyses report signals for improved engagement/abstinence windows and reduced health-care utilization and costs, though interpretation requires standard caution about sponsorship and design heterogeneity [6, 88–91]. In practice, their value proposition is most credible when framed as “between-visit scaffolding”: structured content and reinforcement that help stabilize routines and reduce attrition during the period when medication initiation is most fragile [6, 88–91].

The practical implication for prescribing is not that PDTs “optimize doses,” but that improved engagement during the first weeks of MOUD can support continuity (timely refills, symptom-guided titration, follow-up adherence) and complement risk-triggered outreach and education that clinicians already need to deliver [6, 88–90]. In a workflow sense, PDTs can widen the clinic’s effective reach: they can deliver standardized education and reinforcement consistently, freeing clinician time for complex decisions while also providing signals about engagement that can guide outreach [6, 88–90].

JITAIs broaden this logic by using high-frequency signals – EMA and passive smartphone/wearable streams – as tailoring variables in decision rules that deliver support at high-risk moments while minimizing burden [7, 8, 92, 93]. In addition, negative affect and craving dynamics predict lapses and relapse, and passive features can sometimes identify risk windows when self-report is sparse [94–97]. Hybrid EMA+EHR approaches have reported next-day prediction of non-prescribed opioid use and adherence/retention transitions, but transportability and implementation validity require careful evaluation [98]. The pragmatic near-term role is therefore to support earlier contact rather than to micromanage medication: when risk rises, the safe default is earlier outreach, closer monitoring, and structured reassessment, not algorithmic dose changes [36, 95, 98, 99].

Evidence remains uneven: trials are often pilots with heterogeneous outcomes; missingness is frequently not at random (especially during crisis); and prompts can habituate users unless receptivity is modeled explicitly [8, 92, 99, 100]. The most defensible near-term use of JITAI/personal sensing in MOUD is therefore to prioritize contact and monitoring – earlier check-ins, case management outreach, scheduling adjustments – while keeping dosing/formulation decisions anchored to clinical assessment and guideline thresholds [36, 95, 98, 99]. In addition, governance should treat missingness as a safety signal: sudden disengagement from sensing or EMA may reflect worsening instability, device loss, or avoidance, each of which can warrant outreach rather than punitive interpretations [8, 99, 100].

A related but distinct harm reduction area is respiratory safety technology: consumer-facing monitors can detect desaturation and escalate alerts, and experimental systems explore closed-loop detection paired with naloxone delivery [101–107]. These approaches are promising but remain constrained by limited clinical endpoint trial evidence and challenges of real-world sensor drift, missingness, and polysubstance coexposures [102, 108, 109]. For now, the pragmatic role is adjunctive: detection and alerts complement – rather than replace – human response and broad naloxone access (including OTC naloxone availability) [101, 110]. When used clinically, these tools should be framed as part of a layered safety plan that includes education, naloxone access, and explicit responder routing rather than as a substitute for supervision or clinical follow-up [101, 110].

The evidence hierarchy should remain clear: PDTs, JITAIs, and personal sensing are most defensible as engagement, monitoring, and outreach infrastructure. They are not a mature basis for automated medication selection, methadone or buprenorphine dose changes, or punitive adherence determinations, particularly when missingness, device access, language, and housing instability differ across populations [8, 29, 92–100].

### Tool Class 4: LLM Copilots Best Used as Grounded Conformance Checkers

Clinician-facing LLM copilots can accelerate chart review and generate readable clinical summaries, but their dominant safety failure modes remain hallucinations and omissions [9]. In high-stakes prescribing contexts, the goal should not be “AI writes the plan”; it should be “AI checks the plan” [9]. Domain LLMs can be evaluated for guideline conformance and structured medical reasoning, enabling a “conformance checker” posture rather than autonomous recommendation [111, 112]. WHO guidance emphasizes robust predeployment evaluation, postdeployment monitoring, and explicit accountability for human oversight in LLM-enabled health tools [10]. Runtime mitigation patterns include retrieval-first design, mandatory citations/provenance, and structured self-verification to reduce unsupported statements [113]. In SUD care, this posture is especially important because stigma and uncertainty create fertile ground for automation bias; a copilot that “sounds confident” but is wrong can do disproportionate harm [10, 33, 58].

The safest and most useful pattern is a grounded, retrieval-based copilot that retrieves and cites authoritative sources, exposes its rationale, and flags missing safety steps or guideline conflicts – positioning the tool on the assistive side of the FDA CDS boundary and aligning with HTI-1 evidence-based DSI transparency expectations when embedded in certified health IT [14–16]. For example, copilots can map intake information (withdrawal assessments, last opioid timing, PDMP/UDT patterns, sedative coprescribing) to guideline-anchored decision points and highlight where the proposed induction/titration plan deviates from protocol or omits safeguards (observation windows, sedation checks, naloxone coprescribing, PW fallbacks) [30, 36, 43, 44]. In this role, the copilot’s “output” is less a plan than a set of structured questions and checks that reduce omission risk under time pressure [9, 30].

Concrete safeguards are essential. Clinician-facing copilots should maintain audit trails, model- and knowledge-based versioning, retrieval logs, mandatory source citation for clinical claims, explicit uncertainty or refusal behavior when sources are absent, and clinician sign-off before any clinical action. Safety testing should measure not only factual accuracy but also omissions and unsafe action rates in realistic OUD scenarios, including fentanyl exposure, sedative co-use, PW risk, pregnancy, incarceration transitions, and language-discordant encounters [10, 14–16, 33, 113–116].

Patient-facing conversational agents have a narrower defensible scope [117–123]. Trials and reviews across substances suggest benefits in engagement and motivation with mixed additive effects on core outcomes, and evidence quality varies [117–123]. Given clinical and regulatory stakes, patient-facing agents should remain on the information, skills, and navigation side of the boundary: psychoeducation, motivational support, coping plans, adherence reminders, and warm handoffs to clinicians or services [14, 124]. They should not diagnose SUD, determine MOUD dosing, or manage crises such as overdose risk or acute withdrawal; safety features should include crisis-language detection and explicit escalation to human services [10, 14]. A key practical safeguard is to keep patient-facing outputs generic and support-oriented, while routing individualized decision-making to clinician-facing tools that meet transparency requirements and preserve clinical accountability [10, 14, 16].

## Evaluation and Implementation: What to Measure, How to Deploy, How to Stay Safe

The evaluation problem in AI-supported prescribing is not only discrimination (AUC) but clinical utility, workflow burden, and safety [125, 126]. For predictive models, reporting and appraisal frameworks emphasize data provenance, specification, calibration and uncertainty, external validation, and applicability (TRIPOD+AI; PROBAST+AI) [125, 126]. For AI interventions deployed in workflows, early stage reporting emphasizes human factors and safety signals (DECIDE-AI), while trial reporting frameworks emphasize versioning, human-AI interaction, and dataset shift (SPIRIT-AI; CONSORT-AI) [32, 127, 128]. In SUD prescribing, evaluation must also guard against perverse effects: reduced prescribing may look “safe” in crude metrics while actually reflecting inappropriate restriction of indicated care [59, 74].

The evidence hierarchy should be stated prospectively. EHR CDS for naloxone and protocolized MOUD initiation can be evaluated as implementation interventions with prescribing and safety endpoints; PDMP scores require external validation and fairness review before operational reliance; PDT/JITAI systems require engagement, retention, and implementation validity endpoints; LLM copilots require scenario-based safety and auditability testing before deployment in prescribing workflows; and respiratory technologies require device-specific endpoint evidence before they are treated as clinical safety infrastructure [10, 30–33, 125–132].

In addition, care, calibration, and action thresholds are central [129–131]. Authors and implementers should report calibration-in-the-large, calibration slope, flexible calibration curves with uncertainty, and decision-curve analyses aligned to plausible thresholds, rather than relying on AUC alone [129–131]. External validation should reflect intended practice settings and drift over time, with internal-external cross-validation where feasible and adequate sample sizes for calibration precision [125, 132]. Given the rapid evolution of the opioid supply, threshold choice should be treated as a governance decision tied to explicit clinical intent rather than an arbitrary operating point selected for convenience [33, 125, 132].

Deployment is increasingly standards-based [16, 133, 134]. SMART on FHIR supports EHR-embedded apps; CDS Hooks supports synchronous, workflow-triggered “cards” that return information and one-click actions – structures that also facilitate HTI-1 DSI transparency capture [16, 133, 134]. Governance should set a review cadence (monitor firing and overrides monthly; retune thresholds quarterly; maintain a change-control log linked to safety surveillance) [30, 60, 135]. Post-deployment monitoring and periodic recalibration are essential because data shift is common in medical AI and can silently degrade performance [33, 136]. In prescribing contexts, drift should be interpreted clinically: if the distribution of induction strategies, dosing, or co-prescribing patterns changes abruptly, the system should trigger review even before traditional performance metrics detect a problem [33, 136].

For LLM systems, safety assessment should separate factuality/faithfulness from clinical hazard: the key metric is not just “unsupported claim rate,” but “unsafe action rate” [10, 33]. Scenario-based testing with blinded expert grading and predefined go/no-go thresholds should be part of pre-deployment evaluation, with ongoing monitoring of near misses and overrides [10, 33, 113]. Documentation tools such as model cards and datasheets support auditable disclosure of intended use, known failure modes, subgroup performance, and update cadence – elements that can be mapped onto HTI-1 DSI source attributes [16, 114–116].

## Implementation beyond Advanced US EHR Environments: Europe and Lower Resource Settings

Scalability requires a minimal viable AI strategy. In settings without mature EHR integration, the first step may be a rule-based checklist, registry-derived patient list, pharmacy reconciliation workflow, SMS-supported follow-up protocol, or dashboard that identifies missed appointments and naloxone gaps. These lower complexity tools can still be auditable and equitable if they make their data source, trigger rule, responsible clinician, and next action explicit. In many services, a transparent rule that reliably triggers outreach may be safer and more useful than an opaque model with higher apparent discrimination but weak workflow fit [29–33].

European deployments should account for national variation in opioid agonist treatment models, e-prescribing, pharmacy data, digital identity, patient consent, language, and specialist-versus-primary-care delivery. EHDS may eventually improve data portability and secondary-use governance, but operational AI systems should be locally validated against the data actually available at the point of care. A model trained in a US-integrated delivery system, a Veterans Health Administration dashboard, or a vendor PDMP product should not be presumed transportable to a European clinic, prison health service, rural program, or low-resource setting without recalibration and local safety monitoring [20–29, 64, 65, 125–136].

A practical adoption sequence is therefore: first, digitize the safety-critical workflow; second, add transparent rules and order sets; third, monitor prescribing and retention outcomes; fourth, only then consider predictive modeling when the data, governance, and clinical action pathway are mature enough. This staged approach also makes the manuscript’s recommendations relevant outside well-resourced EHR environments: AI-enabled prescribing can begin with structured human review and simple automation, not only with complex machine learning.

## Equity: Preventing Algorithmic Stigma and Unequal Access

Bias in prescribing-adjacent AI originates from selective coverage, measurement error, proxy features, historical inequities embedded in labels, and drift across sites [74, 137, 138]. PDMP-derived scoring illustrates risk: proxy features (multiple prescribers/pharmacies, travel distance, payment patterns) may correlate with complex or rural care and can become stigma-laden signals if treated as misuse indicators [59, 78, 139]. Without strong external validation, subgroup performance reporting, and explicit guardrails against dispositive use, such scores can contribute to differential access to analgesia or MOUD unrelated to clinical need [59, 74]. In addition, label choice itself can embed stigma: “high risk” may be interpreted as “bad patient,” so systems should emphasize actionable safety needs rather than moralized categories and should explicitly separate risk mitigation from access restriction [59, 74, 78].

Equitable deployment requires layered mitigation [16, 33]. Tools should disclose data gaps (including missing OTP methadone), report subgroup discrimination and calibration, and select thresholds informed by clinical utility and fairness audits rather than convenience [16, 33]. Human-in-the-loop design should require review of underlying evidence and prohibit using scores as the sole basis to initiate, taper, or deny therapy [14, 74]. Monitoring should stratify acceptance, overrides, prescribing changes, and safety outcomes by subgroup, with triggers for recalibration or rollback if disparities worsen [16, 33]. In high-stigma domains, “override patterns” deserve special attention: subgroup differences in overrides may indicate different baseline trust in decision support, different clinician interpretations, or different clinical contexts, each with different ethical implications [16, 33, 59].

Participatory governance strengthens legitimacy and safety: involve people with lived experience and affected communities in explanation design, consent language, and recourse pathways; maintain a standing algorithm governance function linked to quality improvement and compliance [140, 141]; document nondiscrimination policies and appeals pathways; and keep decision support on the “assistive and reviewable” side of the clinical boundary whenever possible [14, 16, 74]. Participation is not only ethical but is operationally useful in designing explanations that reduce disengagement and in defining what constitutes “harm” in real-world care, including harms of denial, delay, or stigmatizing encounters [140, 141].

## Conclusion

Across 2024–2025, AI for OUD-related prescribing and adjunctive addiction care has moved from broad promise to a set of pragmatic tools with distinct evidence levels and governance needs. The most reproducible gains come from transparent, narrowly scoped, workflow-native interventions: risk stratification that routes teams to the very-high-risk tail; naloxone co-prescribing nudges with one-click actions; ED initiation pathways that make standard, high-dose, or low-dose buprenorphine initiation safer and more consistent; and digital therapeutics or JITAI approaches that support early engagement and timely outreach between visits.

The regulatory context is increasingly concrete. US FDA and ONC frameworks clarify device boundaries, transparency, and lifecycle expectations, while European governance adds the EU AI Act, MDR/IVDR medical software qualification, GDPR, EHDS, and HMA-EMA data and AI priorities. Across jurisdictions, these frameworks converge on the same clinical design rule: keep predictive and directive functions transparent and auditable, separate assistive context from device-like recommendations, preserve clinician discretion, and monitor performance after deployment.

Boundaries remain central. PDMP-derived risk scores are advisory and insufficiently validated against hard clinical endpoints; they should not be used as the sole basis to initiate, taper, or deny care. LLM copilots are safest as grounded conformance checkers, not autonomous prescribers. Respiratory safety technologies may complement naloxone access and responder routing but do not replace human response. Across deployments, calibration, drift, equity, subgroup monitoring, documentation, and participatory governance should be planned before scale-up.

The practical path forward is to treat AI as infrastructure for safer, more reliable, and more auditable prescribing. Build on interoperability standards where available, use simpler rule-based supports where infrastructure is limited, prespecify monitoring and change control, and measure success with prescribing-centered outcomes: timely dose optimization, retention at 28/90/180 days, naloxone co-prescribing and fills, and overdose-related safety endpoints. Done well, AI will not supplant clinical judgment; it will make evidence-aligned care easier to deliver, easier to audit, and harder to derail.

## Conflict of Interest Statement

The authors have no conflicts of interest to declare.

## Funding Sources

This study was not supported by any sponsor or funder.

## Author Contributions

Mike Zack: conceptualization – lead, data curation – lead, formal analysis – supporting, project administration – lead, and writing – original draft – equal. Valentin Skryabin: conceptualization – supporting, formal analysis – lead, and writing – original draft – equal.
